# Surface-Catalyzed Secondary Nucleation Dominates the Generation of Toxic IAPP Aggregates

**DOI:** 10.3389/fmolb.2021.757425

**Published:** 2021-11-01

**Authors:** Diana C. Rodriguez Camargo, Sean Chia, Joseph Menzies, Benedetta Mannini, Georg Meisl, Martin Lundqvist, Christin Pohl, Katja Bernfur, Veronica Lattanzi, Johnny Habchi, Samuel IA Cohen, Tuomas P. J Knowles, Michele Vendruscolo, Sara Linse

**Affiliations:** ^1^ Department of Biochemistry and Structural Biology, Lund University, Lund, Sweden; ^2^ Wren Therapeutics Limited, Clarendon House, Cambridge, United Kingdom; ^3^ Centre for Misfolding Diseases, Yusuf Hamied Department of Chemistry, University of Cambridge, Cambridge, United Kingdom; ^4^ Cavendish Laboratory, University of Cambridge, Cambridge, United Kingdom

**Keywords:** self-assembly, amyloid formation, reaction mechanism, optical spectroscopy, peptide purification

## Abstract

The aggregation of the human islet amyloid polypeptide (IAPP) is associated with diabetes type II. A quantitative understanding of this connection at the molecular level requires that the aggregation mechanism of IAPP is resolved in terms of the underlying microscopic steps. Here we have systematically studied recombinant IAPP, with amidated C-terminus in oxidised form with a disulphide bond between residues 3 and 7, using thioflavin T fluorescence to monitor the formation of amyloid fibrils as a function of time and IAPP concentration. We used global kinetic analyses to connect the macroscopic measurements of aggregation to the microscopic mechanisms, and show that the generation of new aggregates is dominated by the secondary nucleation of monomers on the fibril surface. We then exposed insulinoma cells to aliquots extracted from different time points of the aggregation process, finding the highest toxicity at the midpoint of the reaction, when the secondary nucleation rate reaches its maximum. These results identify IAPP oligomers as the most cytotoxic species generated during IAPP aggregation, and suggest that compounds that target secondary nucleation of IAPP could be most effective as therapeutic candidates for diabetes type II.

## Introduction

Currently, approximately 463 million people worldwide have been diagnosed with diabetes which represents about 9% of the world population (Saeedi et al., 2019). Approximately 4.2 million deaths in 2019 were linked to diabetes (Saeedi et al., 2020), directly or indirectly caused by complications in the nerves, kidneys, neurons, visual and cardiovascular systems (Danaei et al., 2014; Huang et al., 2016). The disease is chronic and requires constant treatment and control, at an annual worldwide yearly cost of USD 760 billion (Association, 2018). About 90 percent of the cases involve type II diabetes (Chen et al., 2012), a third of which suffer from late diagnosis aggravating the complications and hampering treatment. The causes of diabetes, especially type 2, are not clear but there is a strong correlation with increasing age, ethnicity, genetics (Andrulionytè et al., 2004; Barroso, 2005; Grant et al., 2009), and obesity (Hutton et al., 1982; Mehnert and Standi, 2000; Knight et al., 2006; Vaiana et al., 2009). Diabetes causes hyperglycemia and insulin resistance (Mehnert and Standi, 2000). As a counter response an overproduction of hormones is initiated. These hormones include insulin and islet amyloid polypeptide (IAPP), which are co-expressed in the pancreas and co-secreted from insulin granules (Johnson et al., 1988; Lukinius et al., 1989). The overproduction of these hormones, in particular IAPP, is linked to the onset of protein aggregation and subsequent dysfunction of the β cells (Janson et al., 1996; Matveyenko and Butler, 2006).

IAPP, also called amylin, is among the most amyloidogenic peptide hormones known (Opie, 1901). In its biologically active form, the peptide is 37 amino acid residues long with amidated C-terminus and an intramolecular disulphide bridge at the N-terminus (Davidson and Hutton, 1987; Sanke et al., 1988; Betsholtz et al., 1989; Hutton, 1989; Mosselman et al., 1989; Nishi et al., 1989; Wang et al., 2001). IAPP aggregates are identified for a majority (90%) of patients with type 2 diabetes and the true prevalence is likely to be even higher due to difficulties in identifying these aggregates in the pancreas (Westermark, 1972; Clark et al., 1988; Betsholtz et al., 1989). Moreover, IAPP aggregation is a major cause of failed β-cell transplantation in treatment of type I diabetes (Westermark et al., 2005). For these reasons, there is considerable interest in targeting IAPP for diabetes treatment. This will require knowledge of the molecular pathways underpinning the aggregation process of IAPP, in particular the correlation between discrete mechanistic steps and the production of toxic species that trigger the pathology.

Studies of the aggregation of other disease-related proteins, such as Aβ (involved in Alzheimer’s disease) and α-synuclein (involved in Parkinson’s disease), have uncovered the critical role of secondary nucleation, whereby the surfaces of fibrillar aggregates catalyse the nucleation of new aggregates from monomers; this leads to the generation of oligomeric intermediate species, which are toxic to neurons (Cohen et al., 2013; Gaspar et al., 2017). In the case of IAPP, the oligomers have been linked to the death of β cells, and the rate of oligomer formation appears to be enhanced by plasma and lipid components (Rodriguez Camargo et al., 2017; Rodeiguez Camargo et al., 2018). These toxic IAPP oligomers also appear to be transiently populated during the aggregation process (Young et al., 2014; Abedini et al., 2016). The existence of a secondary mechanism in the aggregation of IAPP was previously proposed from measurements of Tyr fluorescence anisotropy *versus* time (Padrick and Miranker, 2002). Surface-catalysed secondary nucleation has also been inferred for the proliferation of aggregates of the 10-residue IAPP fragment SNNFGAILSS(Ruschak and Miranker, 2007). However, previous studies have used synthetic IAPP peptide or peptide fragments, and include organic co-solvents such as DMSO and HFIP, which can significantly affect the rate of aggregation (Padrick and Miranker, 2002); typically 1–4% DMSO or HFIP is used, corresponding to 20,000–160,000-fold molar excess relative to 10 µM IAPP. In addition, the overall rate of aggregation observed between studies appears to vary over two orders of magnitude under similar conditions, which might be attributed to variations in the purity and homogeneity of the peptide.

In the present work, we have overcome these previous challenges through the development of a protocol with which the aggregation kinetics can been monitored starting from highly pure monomeric recombinant native human IAPP. The protocol includes the production of recombinant human IAPP peptide to ensure sequence homogeneity, careful control of the initial conditions and quality of the peptide using repeated size-exclusion chromatography for monomer isolation, inertness of the reaction vessels, quiescent conditions, and total absence of organic solvents. Employing these factors has resulted in highly reproducible kinetic data.

To connect the measurements of IAPP aggregation with the underlying microscopic processes, the kinetic data has been subjected to global analysis which identify a minimal set of microscopic steps underlying the overall aggregation process together with their associated rates and significance. This strategy reveals that the IAPP aggregation mechanism is dominated by secondary nucleation of monomers on the fibril surface. INS-1 832/13 rat insulinoma cells were used to investigates the effect, after both short (30 min) and long (24 h) incubation, of samples from different time points of the aggregation process. The results indicate that oligomeric intermediates of IAPP cause cellular dysfunction.

## Results and Discussion

### Size-Exclusion Chromatography Generates Monomeric IAPP

In order to study the microscopic processes involved in the aggregation of IAPP, and to resolve quantitatively the underlying rate constants of the microscopic processes underpinning the aggregation reaction, it is essential to generate highly reproducible experimental measurements of aggregation over a range of peptide concentrations. Through optimising the experimental conditions, including the sample purity, the initial conditions, the inertness of all surfaces involved, the area of all surfaces and the linearity of the reporter used (Hellstrand et al., 2010; Meisl et al., 2016), the recorded reaction profile from such experiments is an accurate reflection of the molecular processes leading from monomers to fibrils, and the data can be analysed to reveal information about these processes. The ability to generate such data has previously been impeded, in particular, by the difficulty in purifying IAPP and isolating the monomeric species from other aggregated species. To overcome these obstacles, we have developed a protocol using successive rounds of size-exclusion chromatography (SEC). Successive rounds of SEC guarantee the quality of the measurements of the aggregation, and allow for subsequent theoretical analysis of the aggregation mechanism, as previously employed for other proteins and peptides, such as Aβ (Cohen et al., 2013).

**FIGURE 1:**
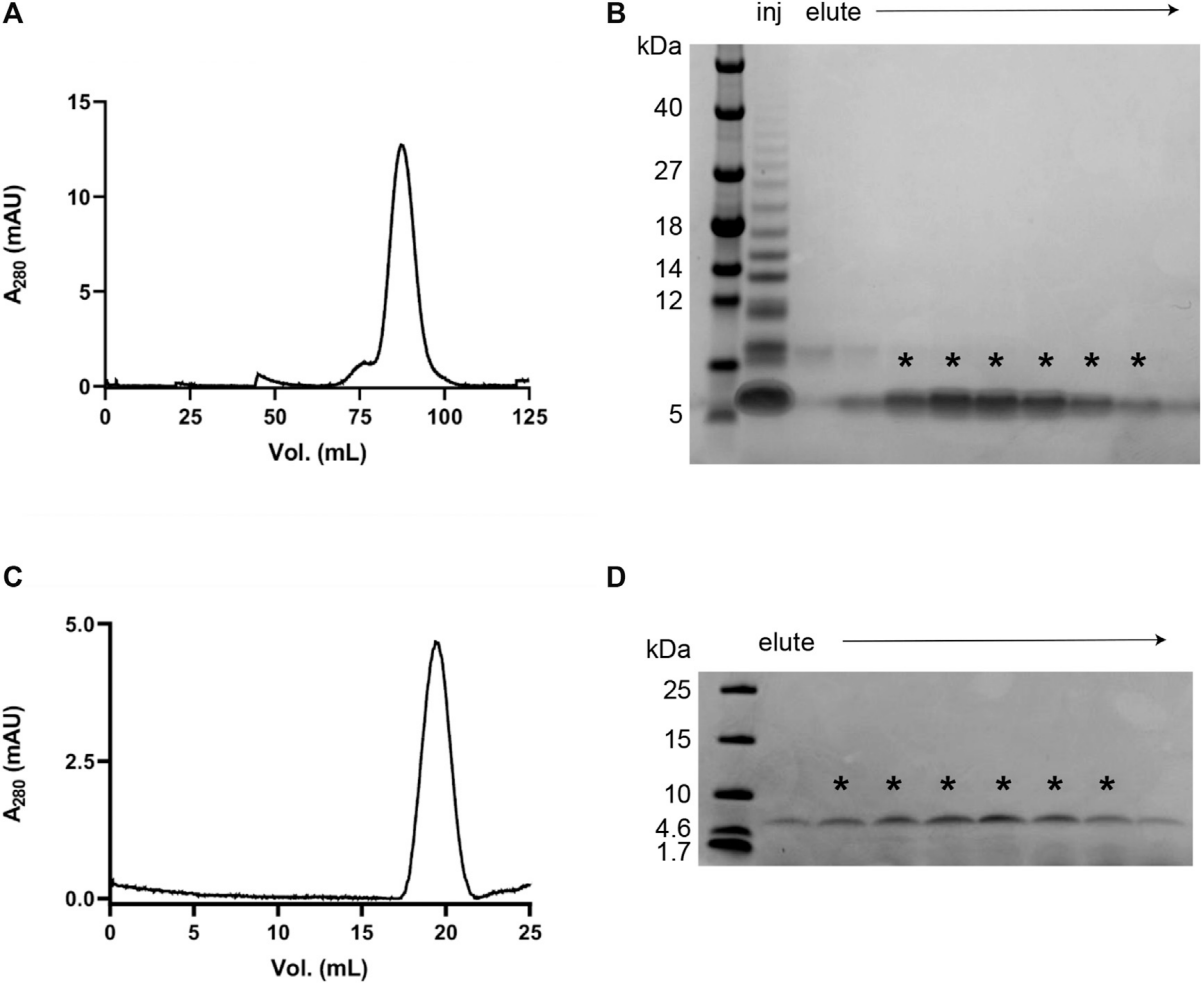
Purification of monomeric recombinant IAPP. **(A)** Chromatogram from a SEC purification using a HiPrep 16/60 Sephacryl S-100 HR column in 35 mM sodium acetate buffer, pH 5.3. **(B)** SDS PAGE using Tris-Tricine gels (10–20% polyacrylamide) of the fractions obtained after SEC as shown in **(A)**. The fractions containing monomeric IAPP (indicated with asterisks) were collected, combined, and lyophilised for another round of SEC. **(C, D)** Chromatogram and SDS PAGE from the second SEC purification using Sephacryl S-100 HR Tricorn 10/300 GL column in 35 mM sodium acetate buffer, pH 5.3.

The protocol was developed by exploring a variety of conditions in terms of buffer composition, pH, temperature and size-exclusion resin in order to produce IAPP monomers without excessive losses of the peptide. We found that a rigid allyl dextran/bisacrylamide matrix (as in the Tricorn 10/300 GL Sephacryl S-100 HR column) has minimal interaction with IAPP, which allows for good separation of the monomer from aggregates and enables isolation of homogeneous samples (Figure 1). An initial HiPrep 16/60 Sephacryl S-100 HR SEC shows the presence of higher-ordered species together with the monomeric IAPP (Figures 1A,B). Pooling the fractions containing monomeric IAPP and subjecting them to another round of size exclusion (using a Tricorn 10/300 GL Sephacryl S-100), an extremely pure monomeric IAPP sample was obtained. With this protocol the initial conditions are well-controlled, and we can study IAPP aggregation kinetics and derive the molecular mechanism of aggregation (Figures 1C,D).

### Reproducible Aggregation Kinetics of Human IAPP

Starting with pure monomeric IAPP samples, with concentrations ranging from 2 to 10 µM IAPP, the aggregation process was studied by monitoring the fluorescence of ThT as a function of time. In the present work, the aggregation process was studied at mildly acidic pH (5.3) at an ionic strength of 183 mM to reflect the physiological environmental conditions inside the β-cells where IAPP is proposed to aggregate initially (Hutton, 1982). All experiments were initiated with a temperature jump from 0 to 37 C.

**FIGURE 2:**
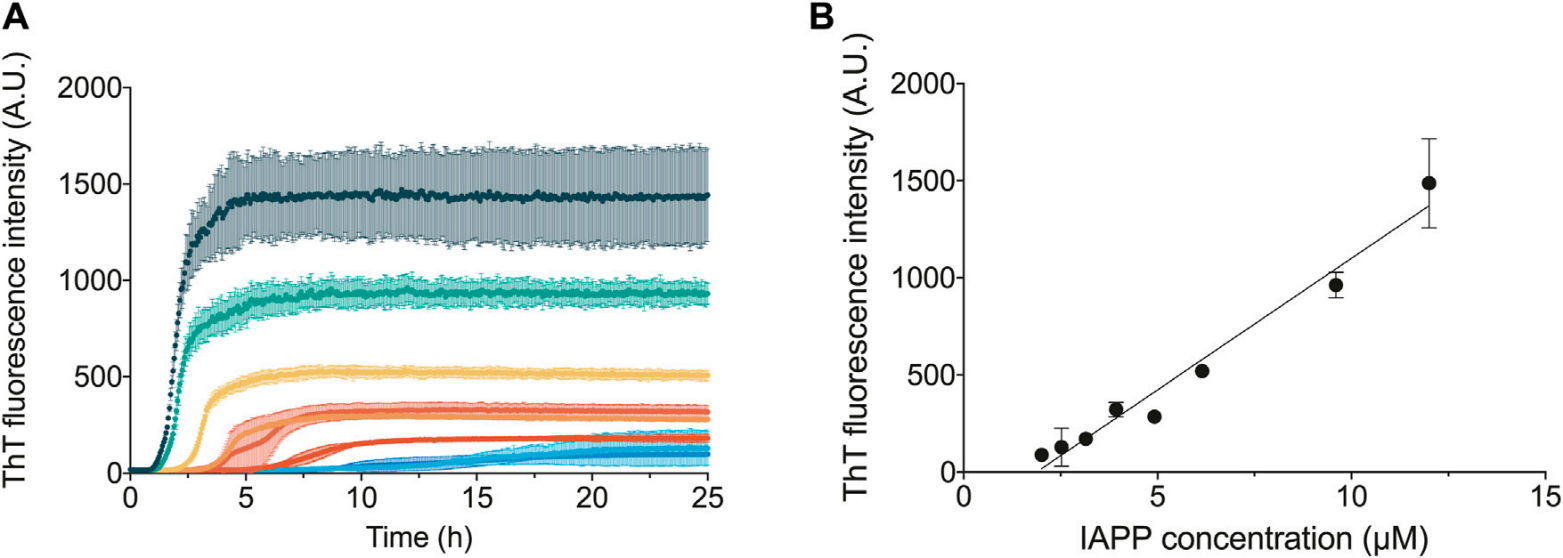
Concentration dependence of IAPP aggregation. **(A)** ThT fluorescence traces following the aggregation of IAPP over time at varying monomer concentrations, which are represented in different colours. **(B)** ThT fluorescence amplitude at the end of the aggregation reaction against the IAPP monomer concentration. All the experiments shown in this figure were carried out in 35 mM sodium acetate buffer, pH 5.3, 150 mM KCl.

The aggregation curves of recombinant IAPP presented here are sigmoidal-like (Figure 2) and qualitatively similar to the data presented in other publications studying the aggregation of IAPP, or shorter fragments of IAPP, using tyrosine anisotropy (Padrick and Miranker, 2002), light scattering (Ruschak and Miranker, 2007) or ANS fluorescence (Kayed et al., 1999). The curves are also qualitatively similar to those observed for non-amidated IAPP (Lundqvist et al., 2021). The ThT fluorescence intensity over time was found to be highly reproducible and dependent on the initial concentration of IAPP monomers in solution (Figure 2). Moreover, both the final fluorescence intensity, as well as the overall rate of aggregation, were found to increase with the concentration of IAPP in a systematic manner (Figure 2).

**FIGURE 3:**
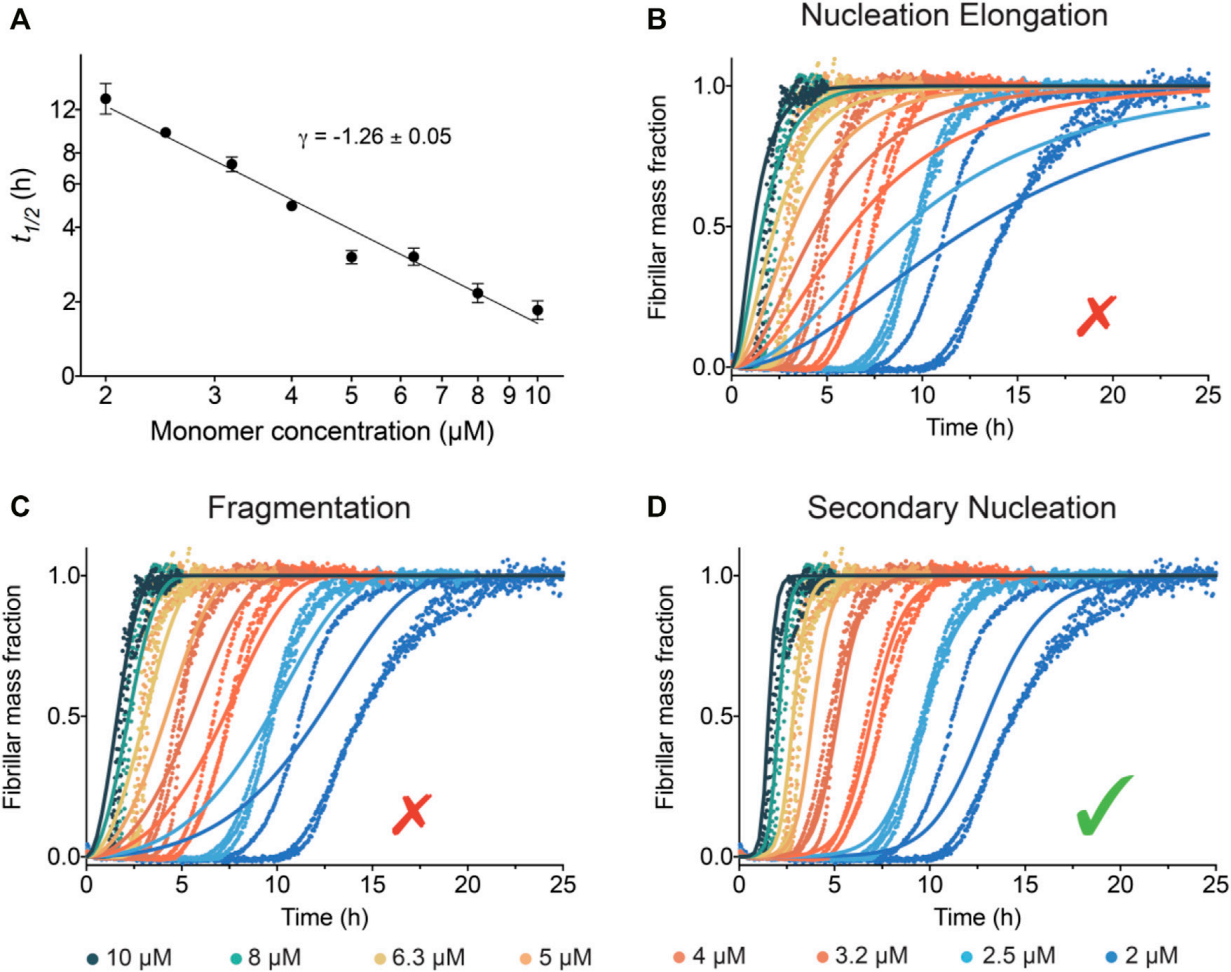
Global kinetic analysis of the IAPP aggregation reactions. **(A)** Double logarithmic plot of the average *t*_*1/2*_ *vs* the initial IAPP monomer concentration. The slope of these points gives the scaling exponent γ. **(B**–**D)** Global fits to the normalised ThT fluorescence intensity as a function of time using models where **(B)** nucleation and elongation occurs with no secondary pathways, **(C)** fragmentation is dominant, and **(D)** secondary nucleation is dominant. Note that the best fit of the IAPP aggregation corresponds to the model where secondary nucleation is dominant.

The half-time of the aggregation process, t_1/2_ (time to formation of half of the final aggregate mass), *versus* initial monomer concentration *[m]*_*0*_ was fitted to a power law, t_1/2_ ∝ *[m]*_*0*_^γ^, in order to quantify the concentration dependence of the aggregation. The scaling exponent γ is related to the reaction order of the dominant nucleation mechanism generating new aggregates (Cohen et al., 2012). The scaling exponent for IAPP is found to be γ ∼ −1.26 ± 0.05 (Figure 3A). The value of this scaling exponent is reproducible between discrete batches of the peptide; however, deviation of the absolute *t*_*1/2*_ values can be observed, which may originate from errors in the estimates of the concentration of IAPP (Supplementary Figure S1). A scaling exponent of approximately −1.2 to −1.3 excludes aggregate proliferation by fragmentation as a dominant mechanism, in which case γ ≈ −0.5 is expected, i.e., a weaker monomer dependence as new aggregates are generated by fibrils alone (Cohen et al., 2012). Moreover, the scaling exponent for IAPP is in the range of that obtained for Aβ42 (*γ* = -1.3), which has previously been shown to aggregate via a surface-catalysed secondary nucleation mechanism (Cohen et al., 2013).

### IAPP Aggregates via a Mechanism Dominated by Surface-Catalysed Secondary Nucleation

The kinetic traces of IAPP aggregation were analysed using the AmyloFit platform (Meisl et al., 2016) to connect the macroscopic measurements of protein aggregation to the microscopic mechanisms using chemical kinetics. The aim of the analysis is to describe the entire set of kinetic data at all different IAPP monomer concentrations using a single rate law (Figures 3B–D). The fitting results for IAPP show that the data are collectively best described by a model in which the main source of new aggregates is a process involving fibril surfaces catalysing the nucleation of IAPP monomers (Figure 3D). Indeed, the results show excellent agreement between this model and the experimental kinetic data (Figure 3D). Conversely, when the data were fitted to other aggregation mechanisms, i.e. nucleation-elongation (Figure 3B) or nucleation-elongation and fragmentation (Figure 3C), they were not well-described by the kinetics models.

**FIGURE 4:**
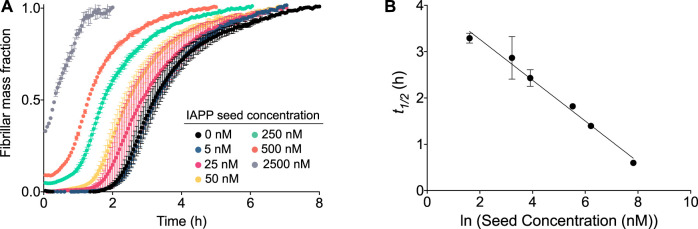
Aggregation of IAPP in the presence of preformed fibrils. **(A)** Kinetic profile of 5 µM IAPP in the absence or presence of increasing concentrations of preformed fibril seeds. **(B)** t_1/2_ of the aggregation as observed from (A) as a function of the logarithm of the seed concentration.

The aggregation mechanism of IAPP was further investigated by aggregation experiments of IAPP in the presence of preformed seeds (Figure 4). For an aggregation process in which secondary nucleation is the dominant process, addition of small amounts (on the order of 1% or less) of preformed fibril seeds will result in a significant reduction of *t*_*1/2*_. This phenomenon is not observed in aggregation processes which include only primary nucleation and elongation, since the elongation of the small quantity of seeds would be within the noise level of the experiment and there would not be a shift in *t*_*1/2*_ (Cohen et al., 2012). As shown in Figure 4, a significant acceleration in the aggregation reaction is observed in the presence of preformed seeds. The acceleration of aggregation increases as a function of the seed concentration (Figure 4). In fact, *t*_*1/2*_ shows a linear dependence on the logarithm of the seed concentration, which is expected of self-seeding behaviour in the presence of secondary pathways (Arosio et al., 2014) (Figure 4B). The results also reveal that even low concentrations of seeds, i.e., 0.5%, are sufficient to accelerate the reaction, which confirms that secondary processes are involved in the aggregation mechanism of IAPP. The elongation rate constant, *k*_*+*_, was estimated by using the initial gradient of the highly seeded data (33%) and the average fibril length determined from cryo-TEM measurements, to be approximately 5^.^10^5^ M^−1^s^−1^ (see materials and methods) (Meisl et al., 2014). Taken together, the experimental and theoretical results reported here reveal unambiguously that IAPP aggregates through a mechanism governed by surface catalysed secondary nucleation.

The pH (5.5) selected for the present study mimics the intragranular pH (5–6) of the insulin-secretory granule, where IAPP is located (Hutton et al., 1982). While some of the IAPP will be secreted from these granules, the data from experiments performed at pH 7.5 (Supplementary Figures SI4, I5) are better fitted if secondary nucleation is included, implying that the mechanism we describe at pH 5.5 extends to neutral pH.

**FIGURE 5:**
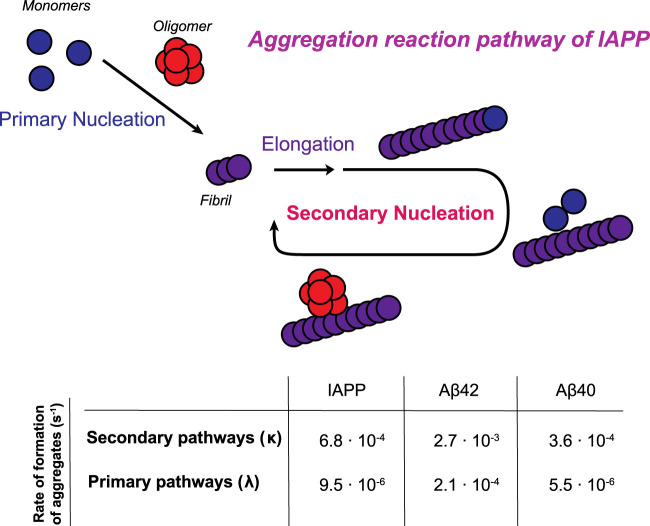
Schematic illustration of the aggregation reaction pathway of IAPP. Monomers initially aggregate through a primary pathway (primary nucleation and elongation). Once a critical amount of fibrils has been formed, the catalytic nature of secondary nucleation becomes the dominant process in generating the oligomers in the aggregation process. In the table, the rates of formation of aggregates through primary and secondary pathways are calculated for a 5 µM sample in 33 mM acetate, 150 mM KCl, pH 5.3. Rate constants of Aβ42 in 20 mM sodium phosphate, pH 8.0 and Aβ40 in 20 mM sodium phosphate, pH 7.4 are obtained from (Cohen et al., 2013; Meisl et al., 2014).

The results for IAPP aggregation presented here can be compared to other peptides that have been shown to aggregate via a mechanism dominated by secondary nucleation (Aβ40, Aβ42 and α-synuclein) (Cohen et al., 2013; Buell et al., 2014; Meisl et al., 2014). The scaling exponent γ of IAPP under native conditions, pH 5.3 and an ionic strength of 183 mM, where its net charge is close to +2 is in the same range as previously detected for Aβ42 (determined under conditions where its net charge is close to -3) (Figure 5). Future quantitative comparison at identical solution conditions and as a function of pH and salt to modulate the influence of electrostatic interactions will provide additional insights into the commonalities and specifics of the aggregation mechanisms among different proteins and peptides.

### IAPP Aggregation Generates Species That Are Toxic to Insulinoma Cells

**FIGURE 6:**
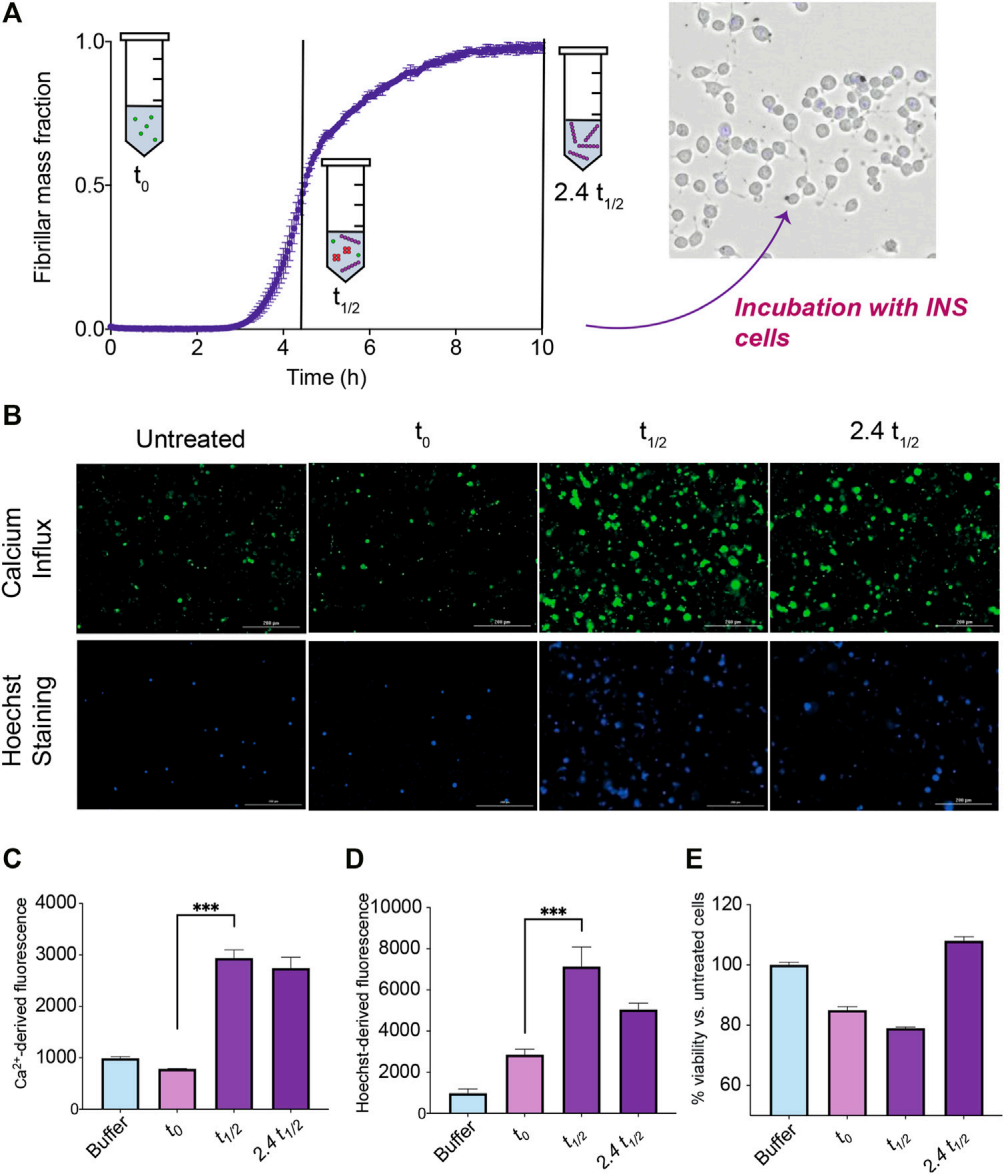
Toxicity measurements of cells exposed to IAPP samples from an ongoing aggregation reaction. **(A)** During the time course of aggregation, samples at the beginning of the reaction where IAPP species are monomeric (*t*_*0*_), at the middle of the reaction where IAPP is ca. half monomeric and half fibrillar and low but significant levels of oligomers are present (*t*_*1/2*_), and at the end of the reaction where samples are mostly fibrillar but some oligomers are still likely to be present (2.4 *t*_*1/2*_), are taken out and incubated with insulinoma cells in order to assess their toxicity. **(B)** Representative images indicating either the fluorescence of Fluo-4 or Hoechst 33,342 of insulinoma cells after treatment with IAPP species at different timepoints. **(C)** Fluorescence intensity of Fluo-4 indicating Ca^2+^ influx levels of cells treated with IAPP species at different timepoints. **(D)** Fluorescence intensity of the apoptotic marker Hoechst 33,342 of cells treated with IAPP species at different timepoints. **(E)** Viability of cells treated for 24 h with IAPP species at different timepoints assessed by means of the CellTiter-Glo assay.

Three different cell biology assays were performed in order to study the biological activity of species generated during the IAPP aggregation reaction. Insulinoma cells were exposed to IAPP samples taken at different time points of the aggregation reaction (Figure 6). These samples included: 1) IAPP at the start of the aggregation process, where it is mostly monomeric (*t*_*0*_); 2) IAPP at the *t*_*1/2*_ of the aggregation process, where there is a significant population of oligomers is expected to coexist with monomers and fibrils; 3) IAPP at the end of the aggregation process, 2.4 *t*_*1/2*_, where there are predominantly fibrillar species (but a smaller fraction of both oligomers and monomers remains) (Michaels et al., 2020). The ability of these samples, from the three different time points in the aggregation time course, to cause cellular dysfunction either by early or late cell toxicity readouts were assessed.

The level by which the samples disrupted cellular membranes and induced Ca^2+^ influx, which is widely regarded as a phenomenon associated with the toxicity of aggregates (Flagmeier et al., 2017), were measured after 30 min of treatment as an early readout. The Fluo-4 calcium indicator was used to measure intracellular calcium levels. A significant increase in the amount of calcium influx for cells exposed to IAPP samples at *t*_*1/2*_ and 2.4 *t*_*1/2*_ were observed (Figure 6C). These results suggest that IAPP aggregation intermediates are able to trigger Ca^2+^ influx within insulinoma cells.

The apoptotic marker Hoechst 33,342 was also used to investigate the 30 min treated cell samples. Interestingly, a significant increase in the signal were observed for the cells treated with the *t*_*1/2*_ sample compared to the *t*_*0*_ sample (Figure 6D). Also the 2.4 *t*_*1/2*_ sample showed an increased signal i.e., the samples containing species other than monomers shows an increase in cytotoxicity after 30 min treatment.

The CellTiter-Glo luminescent assay was used to estimate the viability of the cells by measuring the ATP levels produced by metabolically active cells after a 24 h treatment by the three different samples. A decrease in viability occurred in the cells treated with the *t*_*1/2*_ sample (Figure 6E). The 24 h assay also reveals that the *t*_*0*_ sample also decreased the cell viability, which could be attributed to monomeric IAPP sample aggregating, i.e., producing oligomers, during the course of the 24 h assay.

Taken together, these results show that only the *t*_*1/2*_ samples, where oligomeric species are present at their maximum concentration for a secondary nucleation driven reaction, are able to both trigger significant calcium influx and cause a significant decrease of cell viability.

## Conclusion

The results of this work reveal that the aggregation process of recombinant IAPP under quiescent conditions is dominated by secondary nucleation catalysed by fibril surfaces. Moreover, the oligomeric species generated from secondary nucleation appear to be significantly toxic to insulinoma cells. These results provide a possible rationale for the association between the aggregation of IAPP and the death of β-cells in diabetes type II. This study therefore identifies IAPP oligomers as an important target for drug discovery for diabetes type II, and indicates that the design of inhibitors of secondary nucleation could be developed as a treatment to preserve or restore the function of β-cells in this disease.

## Materials and Methods

### Expression and Purification in E. coli Cells

The recombinant IAPP peptide (KCNTATCATQRLANFLVHSSNNFGAILSSTNVGSNTY) was produced as described previously (Rodriguez Camargo et al., 2015) with the following modifications. All expression media were supplemented with ampicillin (Duchefa Biochemie A0104.0010) to a final concentration of 50 μg/ml. Protein expression was induced by adding IPTG to a final concentration of 0.4 mM. After induction, the cultures were incubated at 20 C whilst shaking at 200 rpm overnight in baffled flasks. Cultures were harvested by centrifugation at 5,000 g. The resulting pellet from 1 L culture was resuspended in 25 ml of 20 mM HEPES, 0.1 mM EDTA, 2 M urea, 50 mM NaCl, pH 8. The eluate, after the chitin step, containing the fusion protein leader-IAPP was concentrated from 100 to 15 ml using 3 kDa cutoff centrifugal filters (Amicon) where after the buffer were changed by using six disposable PD 10 Desalting Columns (GE Healthcare, 17-0851-01) to the standard buffer (20 mM HEPES, 0.1 mM EDTA, 2 M urea, 50 mM NaCl, pH 8.0) in parallel. The lyophilised fraction of IAPP from the HPLC step was dissolved in 35 mM sodium acetate buffer pH 5.3 keeping the concentration of peptide lower than 100 µM and oxidised by the addition hydrogen peroxide to a final concentration of 3 mM and incubated for 6 h at 4 C after which the peptide was again lyophilized.

### Size-Exclusion Chromatography Before Kinetics

The lyophilised powder was dissolved in 5 ml, 8 M urea, 35 mM acetate and pH 5.3 to give an approximate protein concentration of 70 μM, as determined by absorbance spectroscopy using ε_280_ = 1615 L mol^−1^ cm^−1^. The solution was loaded onto a HiPrep 16/60 Sephacryl S-100 HR (17,116,501 GE Healthcare) size exclusion column and proteins were separated using a flow rate of 1.0 ml/min. Fractions containing only monomeric IAPP were combined, yielding 18.6 µM IAPP in 14 ml, aliquoted, lyophilized and stored at -80 C for use in the kinetics experiments.

Just prior to each kinetics experiment, the lyophilised peptide was solubilised in 1 ml, 35 mM sodium acetate, pH 5.3, and loaded onto a Tricorn 10/300™ GL Sephacryl S-100 HR size exclusion column (17,061,201 GE Healthcare) equilibrated in the same buffer. Fractions containing monomeric IAPP were collected, combined, and kept on ice until use in the kinetics experiments.

*Discarded options for size-exclusion chromatography.* Several buffer components (urea, GuHCl), pH values (4.0, 5-0, 5.3, 5.5 and 6.0). NaAc concentrations (from 35 to 1.7 mM), temperatures (above 4 C) and column types [Superdex 30 increase 10/300 GL (GE Healthcare), Superdex 75 increase 10/300 GL (GE Healthcare)] were discarded during initial optimisation of monomer isolation because they lead to loss of more than 80% of the peptide, or the purity of the monomeric sample was compromised.

### SDS Polyacrylamide Gel Electrophoresis

Novex™ 10–20% Tricine Protein Gels, 1.0 mm (Invitrogen™ EC66255BOX) were used for the SDS-PAGE analyses. The gels were run for 45 min at 150 V in Tris/tricine running buffer (0.1 M Tris, 0.1 M tricine, 0.1% SDS, pH 8.25) and stained with InstantBlue® Protein Stain (SKU: ISB1L Expedeon).

### Mass-Spectrometry Analysis

Monomer of oxidized IAPP was isolated using SEC in 30 mM acetic acid pH 5.3, dried under vacuum and dissolved in 4 μl 0.1% Trifluoroacetic acid (TFA), 2% acetonitrile (ACN). Matrix solution, 0.5 µl consisting of 5 mg/ml α-cyano-4-hydroxy cinnamic acid, 80% ACN, 0.1% TFA, were mixed with 1 µl sample and spotted on a MALDI stainless steel plate. MS spectra were acquired using a Autoflex Speed MALDI TOF/TOF mass spectrometer (Bruker Daltonics, Bremen, Germany) in positive reflector or positive linear mode. The observed mono-isotopic mass of 3,900.9 Da corresponds to full-length oxidized and amidated IAPP.

### Aggregation Kinetic Assay

All samples were prepared in low-binding tubes (Axygen) on ice. Monomeric IAPP in 35 mM acetate buffer pH 5.3 was mixed with a 2.5 M stock solution of KCl and a 2 mM stock solution of ThT (CalBiochem, purchased from Sigma-Aldrich, Product code 596,200, dissolved in H_2_O and filtered through a 200 nm filter and concentration determined using absorbance) to a final concentration of 33 mM acetate, 150 mM KCl, pH 5.3, 20 µM ThT. Solutions were pipetted into wells of a 96-well Half Area Black/Clear Flat Bottom PEGylated Polystyrene plate (Corning® 3,881), 80 µl per well in triplicates. The ThT fluorescence was monitored through the bottom of the plate over time as a reporter of the amount of aggregates formed using a plate reader (FLUOstar Omega, FLUOstar Galaxy or FLUOstar, BMG Labtech) equipped with 440 nm excitation filter and 480 nm emission filter.

### Seeded Aggregation Assays

Fibril seeds were prepared by adding a 5 µM IAPP solution in 33 mM acetate, 150 mM KCl, 20 μM ThT, pH 5.3, in the 96-well plate and incubating the solution in the plate reader as described above. The ThT fluorescence was monitored to ensure that the aggregation reaction was complete before collecting the seeds. The collected fibril seeds were added to monomer IAPP solutions to final seed concentrations ranging from 0 to 33% of the monomer concentrations (in monomer equivalents). The solutions were pipetted in triplicate to the 96-well plates and the aggregation process was followed by monitoring the ThT fluorescence in the plate reader at 37 C under quiescent conditions, as described above.

### Integrated Rate Law

All analyses involving the determination of the midpoint of the aggregation reaction (*t*_*1/2*_) and the global analysis of the kinetic data were performed using the online Amylofit platform (Meisl et al., 2016). For the global analysis of the kinetic data the following rate law was used, which describes the IAPP species distribution over time and allows for the inclusion of secondary nucleation:

$$\frac{{\left[M\right]}_{t}}{{\left[M\right]}_{\infty }}=1-\left(1-\frac{{\left[M\right]}_{0}}{{\left[M\right]}_{\infty }}\right)e^{-k_{\infty }t}\cdot {\left(\frac{B_{-}+C_{+}e^{\varkappa t}}{B_{+}+C_{+}e^{\varkappa t}}\cdot \frac{B_{+}+C_{+}}{B_{-}+C_{+}}\right)}^{\frac{k_{\infty }^{2}}{{\bar{k}}_{\infty }\varkappa }}$$

where the following definitions are used:

$$\kappa =\sqrt{2{\left[m\right]}_{0}k_{+}{\left[m\right]}_{0}^{n_{2}}k_{2}}$$

$$\lambda =\sqrt{2k_{+}k_{n}{\left[m\right]}_{0}^{n_{c}}}$$

$$C_{\pm }=\frac{k_{+}{\left[P\right]}_{0}}{\kappa }\pm \frac{k_{+}{\left[M\right]}_{0}}{2{\left[m\right]}_{0}k_{+}}\pm \frac{\lambda^{2}}{2\kappa^{2}}$$

$$k_{\infty }=2k_{+}{\left[P\right]}_{\infty }$$

$${\bar{k}}_{\infty }=\sqrt{k_{\infty }^{2}-2C_{+}C_{-}\kappa^{2}}$$

$$B_{\pm }=\frac{k_{\infty }\pm {\bar{k}}_{\infty }}{2\kappa }$$

and where ${\left[m\right]}_{0}$ is the initial concentration of soluble monomers, ${\left[M\right]}_{0}$ and ${\left[M\right]}_{\infty }$ are the mass concentration of fibrils at the start and the end of the reaction respectively, and ${\left[P\right]}_{0}$ and ${\left[P\right]}_{\infty }$ are the number concentration of aggregates at the start and end of the reaction respectively; $n_{c}$ and $n_{2}$ are the reaction orders relative to the monomer of the primary and secondary nucleation pathways respectively; $k_{+}$ , $k_{2}$ , and $k_{n}$ are the rate constants for elongation, secondary nucleation, and primary nucleation, respectively. The equations derived for models with fragmentation, have been described previously (Meisl et al., 2016).

### Cryo-Electron Microscopy

Fibrils were collected after reaching the plateau phase in the aggregation process and analysed by cryogenic transmission electron microscopy (TEM). The samples were loaded as a liquid film on a lacey carbon filmed cooper grid (01881F Lacey F/C, 200 mech Cu; PELCO No.160). A layer of sample less than 300 nm thick was produced on the grid by blotting the extra liquid away at the back of the grid using a filter paper, followed by flash freezing the grid in liquid ethane and whereafter it was stored in liquid nitrogen. The grid preparation was carried out in a controlled environment vitrification system to ensure the stable temperature and humidity in order to maintain the original state of the sample. Images were recorded using a 120 kV electron microscope (Philips CM120 BioTWIN Cryo) with a CCD camera. The size of the fibrils, for statistical purposes, were analysed using ImageJ (Schneider et al., 2012).

### Determination of Elongation Rate Constant

The estimation of the elongation rate constant was performed as described previously (Meisl et al., 2014, 2017). Firstly, the fibril sizes were estimated from length and thickness measurements based on cryo-EM images (Supplementary Figure S6). The average length was determined to be 900 ± 400 nm, and the cross area 80 ± 20 nm^2^. Assuming a protein density of 1.3 g/ml and the molecular mass of 3,906 Da for the IAPP monomer, each fibril was estimated to consist of approximately 14,400 monomers. Subsequently, using the results of the strongly seeded aggregation (33% seeds), the initial gradient, ${dM/dt/}_{t=0}=2k_{+}m\left(0\right)P\left(0\right)$ was derived. To estimate the number of seed fibrils, P (0), the mass concentration of seed fibrils (in monomer equivalents), M (0), which is known, was divided by the number of monomers per seed fibrils. The elongation rate constant was then determined to be approximately 5⋅10^5^ M^−1^s^−1^, within a factor of 3, based on the heterogeneous length and thickness distribution in the fibril samples.

### Cell Cultures

INS-1 832/13 rat insulinoma cells (Merck KGaA, Darmstadt, Germany) were cultured in RPMI-1640 (Thermofisher, United Kingdom) supplemented with 2 mM l-glutamine, 1 mM sodium pyruvate, 10 mM HEPES, 0.05 mM β-mercaptoethanol and 10% heat-inactivated fetal bovine serum. The cell cultures were maintained at 37 C in a 5.0% CO_2_ humidified atmosphere and grown until 80% confluence for a maximum of 20 passages.

### Cell Viability Assay

Cell viability was measured using the CellTiter-Glo Luminescent Cell Viability Assay (Promega) according to the manufacturer’s instructions. Briefly, the cells were plated into a white opaque 96-well plate and treated for 24 h with samples containing 5 µM IAPP from an aggregation time course (6 replicates per condition). The final concentration of IAPP was 1.25 µM (monomer equivalent). Luminescence values were measured using a plate reader (ClarioStar Omega BMG Labtech, Aylesbury, United Kingdom), and cell viability was expressed as a percentage vs untreated cells (taken as 100%).

### Calcium Release Assay

The cytosolic calcium ion (Ca^2+^) levels were measured by exposing the INS-1 832/13 cells loaded with 2.0 μM Fluo4-AM to samples containing 5 µM IAPP taken at different time points of an aggregation reaction (3 replicates per time-point). The final concentration of IAPP was 2.5 µM (monomer equivalent). The emitted fluorescence was recorded after excitation at 488 nm using the fluorescence microscope Cytation5 Cell Imaging Reader and quantified by means of the Gen5 Data Analysis software (BioTek Instruments, Winooski, VT).

### Hoechst 33,342 Staining Assay

INS-1 832/13 cells were treated with samples containing 5 µM IAPP from an aggregation reaction (3 replicates per time-point). The final concentration of IAPP was 2.5 µM IAPP. Cells were stained with the apoptotic marker Hoechst 33,342. The emitted fluorescence was recorded after excitation at 350 nm using the fluorescence microscope Cytation5 Cell Imaging Reader and quantified by means of the Gen5 Data Analysis software (BioTek Instruments, Winooski, VT).

### Statistical Analysis

For cellular assays, comparisons between groups were performed using one-way ANOVA followed by Bonferroni’s multiple comparison test. A *p*-value lower than 0.05 was considered statistically significant.

## Data Availability

The raw data supporting the conclusions of this article will be made available by the authors, without undue reservation.
