# Supplementary material for: Surface-Catalyzed Secondary Nucleation Dominates the Generation of Toxic IAPP Aggregates
Source: Front Mol Biosci. 2021 Nov 1;8:757425. doi: 10.3389/fmolb.2021.757425 (PMC8591229; doi:10.3389/fmolb.2021.757425)
Supplement: Supplementary file 1 [file DataSheet1.pdf]

Supplementary Information

Surface-catalyzed secondary nucleation dominates the generation of toxic IAPP aggregates

Diana C. Rodriguez Camargo[1,2*], Sean Chia[2,3*], Joseph Menzies[2], Benedetta Mannini[2,3], Georg Meisl[3], Martin Lundqvist[1], Christin Pohl[1], Katja Bernfur[1], Veronica Lattanzi[1], Johnny Habchi[2], Samuel Cohen[2], Tuomas PJ Knowles[3,4], Michele Vendruscolo[3], Sara Linse[1&]

1. Department of Biochemistry & Structural Biology, Lund University, P.O. Box 124, SE-221 00 Lund, Sweden
2. Wren Therapeutics Limited, Clarendon House, Clarendon Road, Cambridge CB2 8FH, UK
3. Centre for Misfolding Diseases, Department of Chemistry, University of Cambridge, Cambridge CB2 1EW, United Kingdom
4. Department of Physics, Cavendish Laboratory, Cambridge CB3 0HE, United Kingdom

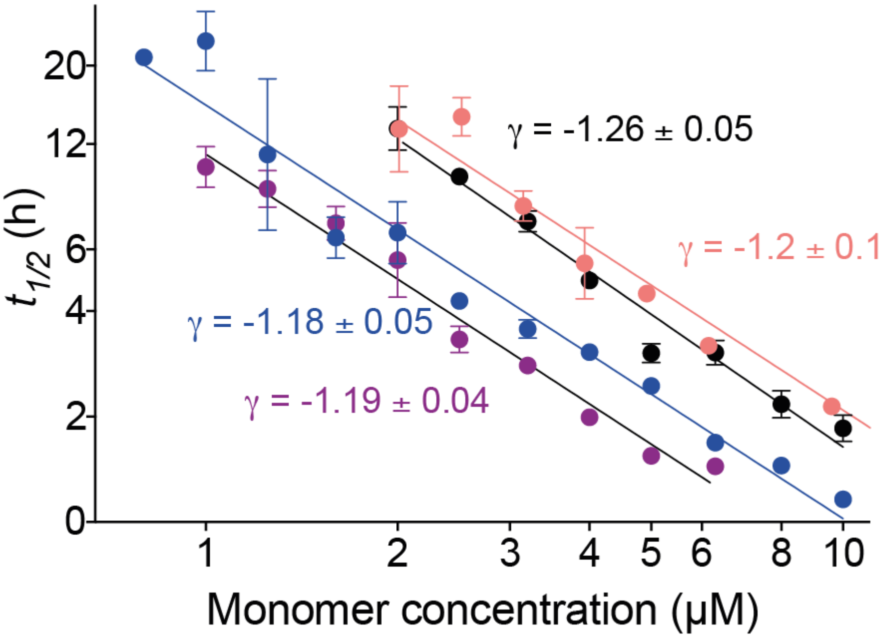

SI Figure 1: Scaling exponent γ from independent experiments. Note that despite a slight difference in the absolute $t_{1/2}$ due to errors in estimating the concentration spectrophotometrically, the scaling exponent γ does not change, thus reflecting the purity of the peptide and the reproducibility of the experiment.

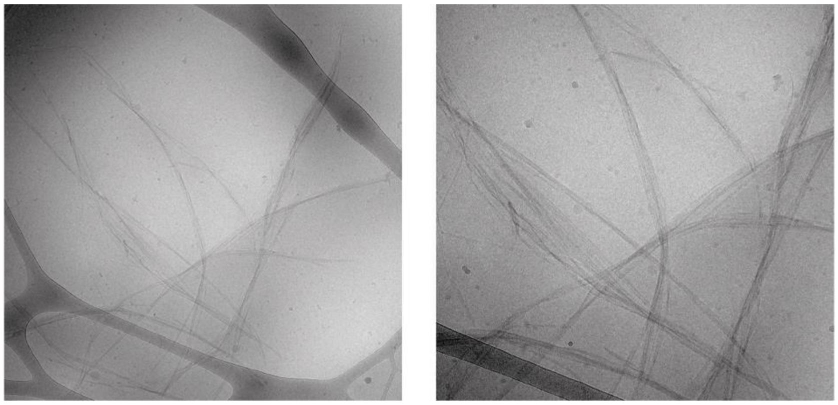

Number of monomers per fibril	k_+ ($M^{-1}s^{-1}$)
14400	$5 \cdot 10^5$

SI Figure 2: Representative cryo-TEM images of fibrils of IAPP and an estimation of the fibril size and rate constant of elongation as calculated from cryo-TEM images and heavily seeded data (Fig. 4).

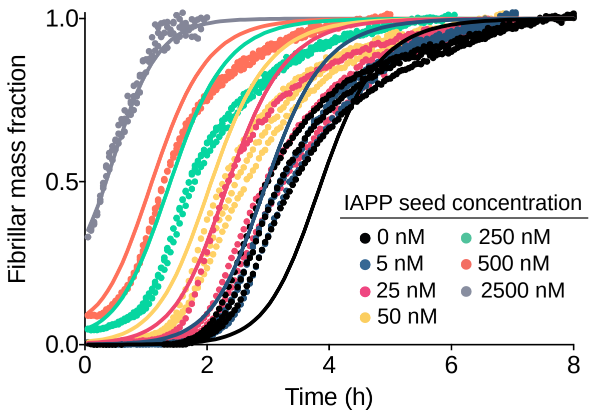

SI Figure 3: Kinetic profile of 5 µM IAPP in the absence or presence of increasing concentrations of preformed fibril seeds (represented in different colours) as shown in Fig. 4A. Solid lines show the predicted curves using primary and secondary nucleation rate constants as derived from the fits in Fig. 3D, and the estimated elongation rate constant using TEM data.

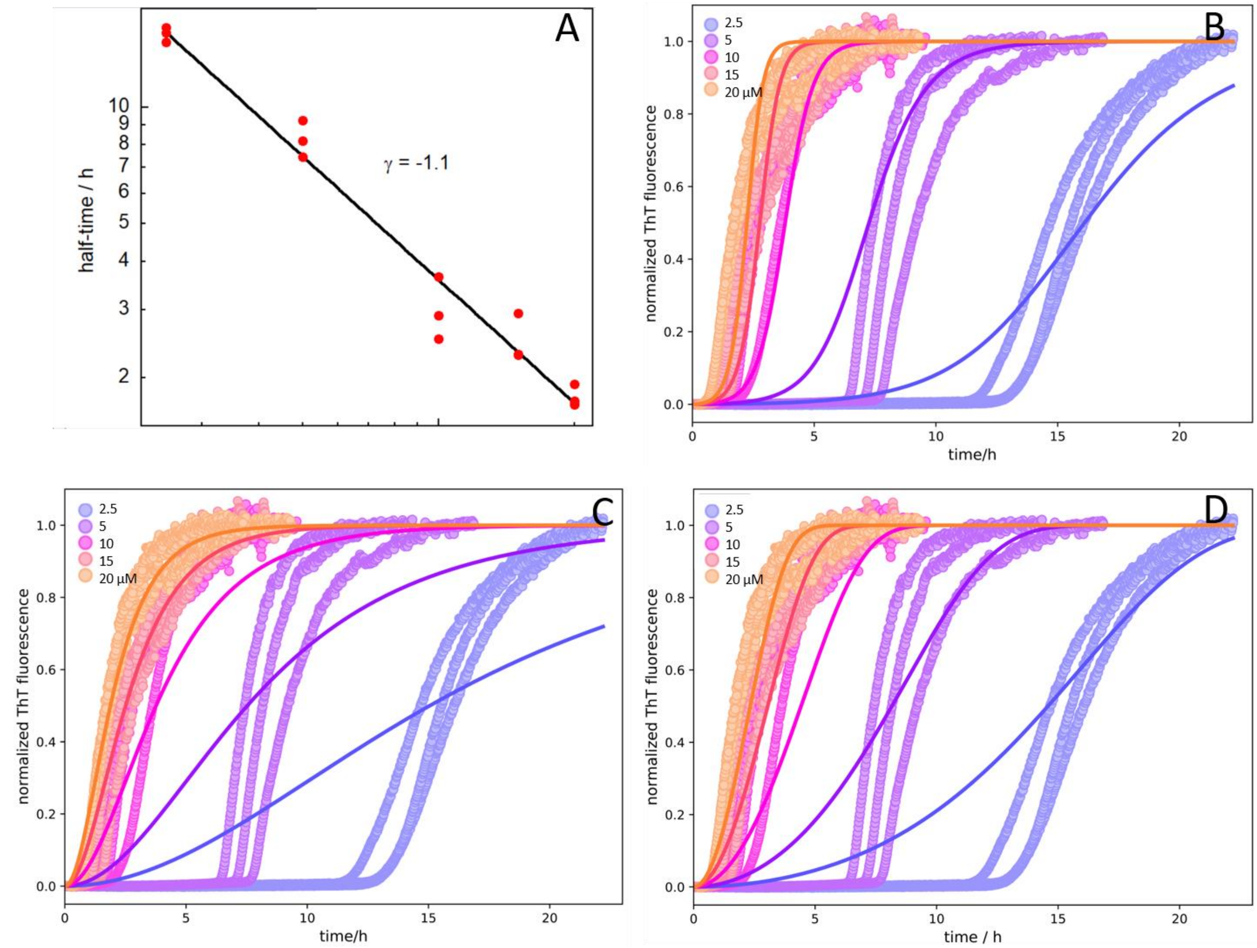

SI Figure 4: Aggregation kinetics of IAPP in 50 mM Tris/HCl,0.2 mM EDTA, 0.02% NaN_3, pH 7.35, 37°C. For this experiment, the peptide was produced using the same protocol as in the main text, resulting in an oxidized peptide with amidated C-terminus. **B-D**) Normalized ThT fluorescence intensity versus time for 3 replicates at each concentration. **A**) Half time of aggregation versus initial monomer concentration The solid line is a fitted power function with exponent γ=-1.1. **B**) Best fit using a model including primary nucleation, multi-step secondary nucleation and elongation. **C**) Best fit using a model including primary nucleation and elongation. **D**) Best fit using a model including primary nucleation, fragmentation and elongation. In all models the reaction orders for primary and secondary nucleation were kept fixed at 2.0 as in the analysis of data obtained at pH 5.5 (main text).

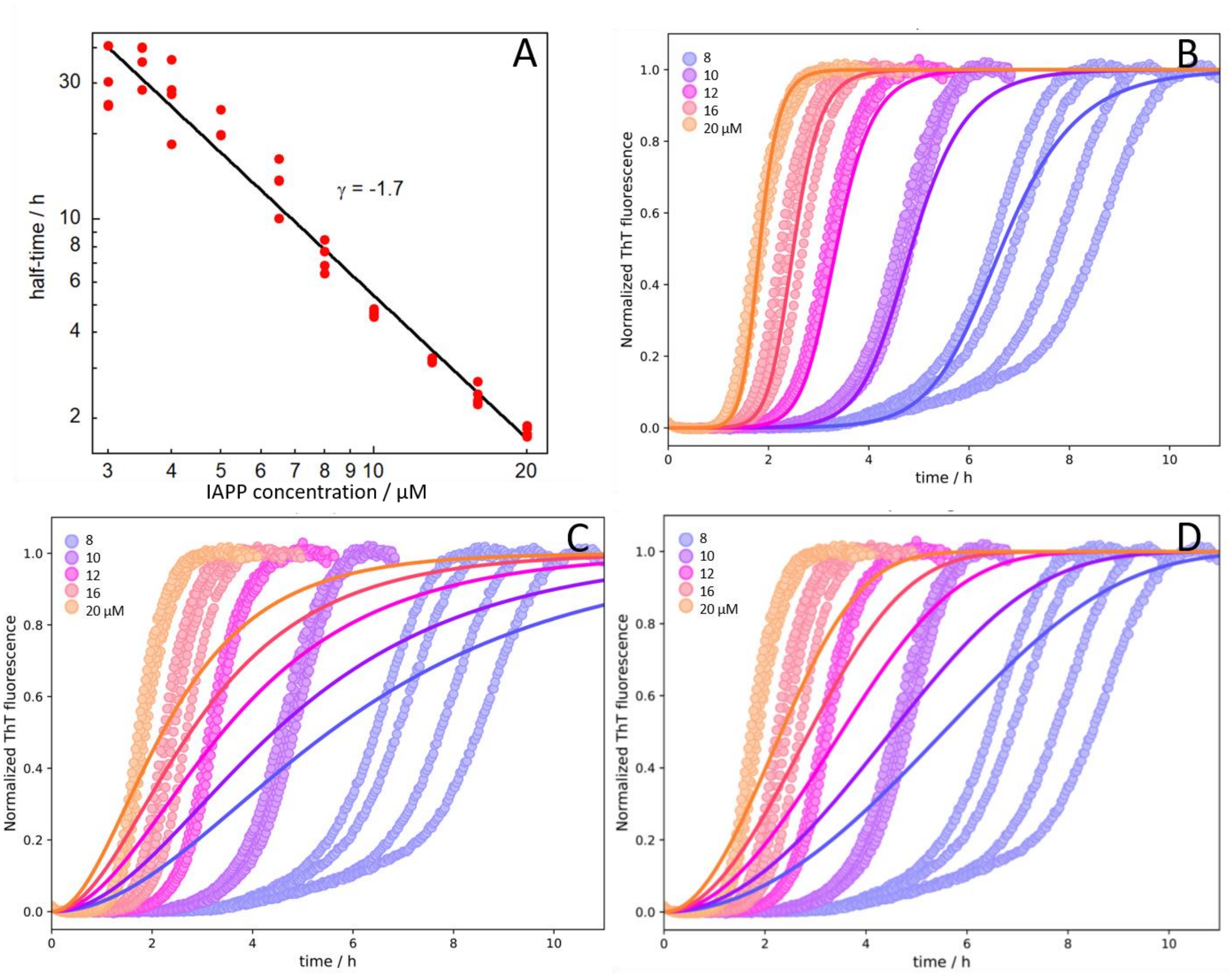

SI Figure 5: Aggregation kinetics of IAPP in 20 mM Tris/HCl, 100 mM NaCl, pH 7.5, 37°C. For this experiment, the peptide was produced using the auto-cleavable tag Npro in the form of its EDDIE mutant (Lundqvist 2021), resulting in an oxidized peptide with C-terminal carboxylate group. **A**) Half time of aggregation versus initial monomer concentration. The solid line is a fitted power function with exponent γ=-1.7. **B-D**) Normalized ThT fluorescence intensity versus time for 4 replicates at each concentration. **B**) Best fit using a model including primary nucleation, secondary nucleation and elongation. **C**) Best fit using a model including primary nucleation and elongation. **D**) Best fit using a model including primary nucleation, fragmentation and elongation. In all models the reaction orders for primary and secondary nucleation were kept fixed at 2.0 as in the analysis of data obtained at pH 5.5 (main text).

SI Table 1: Fitted parameters in the secondary nucleation model as used in Fig. 3D and SI Fig. 3.

Fitted parameter	Fitted value
n_c	3
n_2	1.6
k_+k_n / $M^{-nc}h^{-2}$	$2.2 \cdot 10^{12} \pm 2 \cdot 10^{11}$
k_+k_n / $M^{-(n2-1)}h^{-2}$	$1.4 \cdot 10^{14} \pm 1 \cdot 10^{13}$
